# Viral Interactions and Pathogenesis during Multiple Viral Infections in *Agaricus bisporus*

**DOI:** 10.1128/mBio.03470-20

**Published:** 2021-02-09

**Authors:** Edward Dobbs, Greg Deakin, Julie Bennett, Caoimhe Fleming-Archibald, Ian Jones, Helen Grogan, Kerry Burton

**Affiliations:** a NIAB-EMR, Kent, United Kingdom; b Horticulture Development Department, Teagasc Research Centre, Ashtown, Ireland; c University of Reading, School of Biological Sciences, Reading, United Kingdom; CEH-Oxford

**Keywords:** *Agaricus bisporus*, multiple-virus infection, plus-strand RNA virus

## Abstract

Viral interactions during multiple viral infections were examined in *Agaricus bisporus* cultures harboring 9 viruses (comprising 18 distinct viral RNAs) by statistically analyzing their relative abundance in fruitbodies. Four clusters of viral RNA were identified that suggested synergism and coreplication. Pairwise correlations revealed negative and positive correlations between clusters, indicating further synergisms and an antagonism involving a group containing a putative hypovirus and four nonhost ORFan RNAs (RNAs with no similarity to known sequences) possibly acting as defective interfering RNAs. The disease phenotype was observed in 10 to 15% of the fruitbodies apparently randomly located among asymptomatic fruitbodies. The degree of symptom expression consistently correlated with the levels of the multipartite virus AbV16. Diseased fruitbodies contained very high levels of AbV16 and AbV6 RNA2; these levels were orders of magnitude higher than those in asymptomatic tissues and were shown statistically to be discretely higher populations of abundance, indicating an exponential shift in the replicative capacity of the virus. High levels of AbV16 replication were specific to the fruitbody and not found in the underlying mycelium. There appeared to be a stochastic element occurring in these viral interactions, as observed in the distribution of diseased symptoms across a culture, differences in variance between experiments, and a number of additional viruses undergoing the step-jump in levels between experiments. Possible mechanisms for these multiple and simultaneous viral interactions in single culture are discussed in relation to known virus-host regulatory mechanisms for viral replication and whether additional factors could be considered to account for the 1,000-fold increase in AbV16 and AbV6 RNA2 levels.

## INTRODUCTION

During viral infection, numerous and complex virus-host interactions occur, including appropriation of host machinery and induction of host antiviral defense mechanisms. Further interactions, virus-virus, can result from double and triple virus infections, such as synergistic effects on disease symptom development (1–4). However, the complexity of interactions is massively increased in multiple viral infections where a host harbors large numbers of viruses, a condition that appears to be widespread in some perennial plants and fungi. For example, the grapevine Vitis vinifera harbors large numbers of mycoviruses and plant viruses (5, 6), and there are numerous reports of multiple viral infections in fungi, such as 21 virus-like sequences in a hypovirulent strain of the plant pathogen *Sclerotium rolfsii* (7), 11 viruses in a single isolate of Beauveria bassiana (8), 16 diverse viruses in *Fusarium poae* (9), and 26 distinct viral RNAs in a single isolate of *Agaricus bisporus* (10). Furthermore, deep sequencing technology and bioinformatic analyses of publicly available transcript databases are likely to further increase the number of viral observations in fungi (11).

Multiple viral infections are often asymptomatic or benign or at least have a low fitness cost to the host. However, specific viruses within mixed infections have been associated with a phenotype as pathogenic, changing host behavior by reducing a host’s virulence or stabilizing a coinfection, for instance, in *Cryphonectria parasitica* (4, 12). Some plant diseases occur only upon coinfection, while singular infections are relatively benign, e.g., rice tungro (13), maize lethal necrosis (14), and cowpea stunt disease (15). Virus levels within multiple virus infections can be dynamic over time with variable viral profiles influenced by host, virus-virus, or environment factors, which all can affect pathogenicity (3, 16). Synergistic and antagonistic viral interactions have been associated with changes in viral pathogenesis and their evolution and spatial separation in plant tissues. The outcome of multiple infections can be difficult to predict (17–19), for instance, with the activation or suppression of RNA silencing and the formation of aggregated multiple viral genome structures producing collective infectious units, both having the potential for synergistic and antagonistic virus-virus interactions (20, 21).

The fungus *Agaricus bisporus* is cultivated commercially to produce white button mushroom fruitbodies, a high-value crop. Thirty-nine nonhost RNAs of probable viral origin have been identified in *A. bisporus*. Three viral diseases of *A. bisporus* are economically important due to yield loss or quality loss. La France disease has symptoms of slow mycelial growth and distorted, delayed, or massively reduced fruitbody emergence and is caused by the particulate virus AbV1, consisting of 6 RNAs and 3 associated RNAs (22). Brown cap mushroom disease (BCMD) has symptoms of uniform to variable fruitbody cap browning, a significant quality-loss feature (23), and mushroom virus X (MVX), or Patch disease, has symptoms similar to those of La France disease, with massive yield loss (as nonproductive patches) and distorted mushrooms but the absence of AbV1 virus (24). BCMD and Patch disease were first observed and described at a similar time in the 1990s but often at different locations. The name MVX was originally used to describe both of these emerging diseases, but they are now considered distinct. Fruitbodies of both diseases contain multiple viral infections, and 30 RNAs have been sequenced, consisting of 18 viruses and 8 nonhost ORFans (RNAs with no similarity to known sequences) (10). Sequence comparison suggests that many of these viruses and ORFans are common for both diseases despite initially disparate identification based on gel separation. Gel-separated band H1 is equivalent to the hypovirus-like AbV2, band H3 is equivalent to ORFan2, and bands 18, 19, 22, and 23 are equivalent to AbV16 RNAs 2, 1, 3, and 4, respectively (10, 24). Mushroom bacilliform virus (MBV) has been characterized and identified in the deep-sequenced RNAs of mushrooms with BCMD symptoms and also occurs as a coinfection with AbV1 in La France disease (10, 25).

RNA sequencing of fruitbody samples with symptoms of BCMD showed the highest number of viral RNAs (17–24), while asymptomatic fruitbodies had only 10 to 17 viral RNAs. Nondiseased control samples had 8 viral RNAs (10). Three viruses (AbV2, AbV6, and AbV10) and 5 ORFans (ORFan2, ORFan3, ORFan4, ORFan5, and ORFan7) were present in all 10 samples and, thus, were described as ubiquitous (10). The most abundant viral RNAs in the BCMD samples by depth of coverage were AbV16 and ORFan8. Two viruses were multipartite, AbV6 and AbV16. AbV6 has two component RNAs, each with a common 226-base motif in the 3′ untranslated region (UTR), AbV6 RNA1, encoding a replicase domain, and AbV6 RNA2, encoding a capsid-like motif (10). AbV16 has four component RNAs, each with a common 25-base motif in the 3′ UTR (AbV16 RNA and RNA2, encoding a replicase and methyltransferase, respectively) and belongs to the proposed family *Ambsetviridae* with homologs from plants, fungi, and metagenomic samples (10, 26, 27). Levels of AbV16 RNAs and ORFan8 were massively more abundant (10^3^ to 10^4^ times greater) in the symptomatic brown mushrooms than neighboring white mushrooms from the same infected crop (23). The transition of virus levels, low to very high, occurs early in fruitbody development, coincident with elevated cell division and differentiation (23).

Previous investigations into virus-virus interactions have largely involved binary or lower-order interactions and have not considered multiple virus infections. It is not known how large numbers of viruses in a tissue interact and compete for the host cells’ resources and how adaptive strategies for low-level viral coexistence adopted by some member viruses are affected by the high replicative activity of others. In this study, viruses from an *A. bisporus* strain showing pathogenic symptoms were introduced to an established mycelium of *A. bisporus* harboring ubiquitous viruses, and the subsequent viral interactions were observed through the analysis of viral abundance levels. A variety of statistical analysis and modeling approaches were used to examine virus-virus interactions in fruitbodies displaying disease symptoms and in underlying mycelium. This is the first time the interactions among such a large number of coinfecting viruses have been studied.

## RESULTS

Fully colonized mycelia of *Agaricus bisporus* strain (A15), growing on compost and harboring ubiquitous viruses and ORFans, were infected with additional viruses and ORFans from *A. bisporus* strain MVX-4569. This resulted in the production of brown symptomatic mushrooms (color values, 9.5 to 20.8) among a prevalence of white asymptomatic mushrooms (color values, 7.7 to 8.6). A preliminary experiment was carried out to determine how many of the 30 viral RNAs identified by Deakin et al. (10) were present in MVX-4569-infected mushrooms using quantitative PCR (qPCR) of a single brown mushroom and a single white mushroom from each experiment. This revealed the presence of 18 viral RNAs, comprising 9 distinct viruses, including the multipartite AbV6 (2 segments) and AbV16 (4 segments) and 5 nonhost RNAs, which, per Deakin et al. (10), we term ORFans. The abundances of these 18 viral RNAs were measured by qPCR in the mushroom samples (40 samples from experiment 1 and 49 from experiment 2).

### Viral clusters.

**FIG 1:**
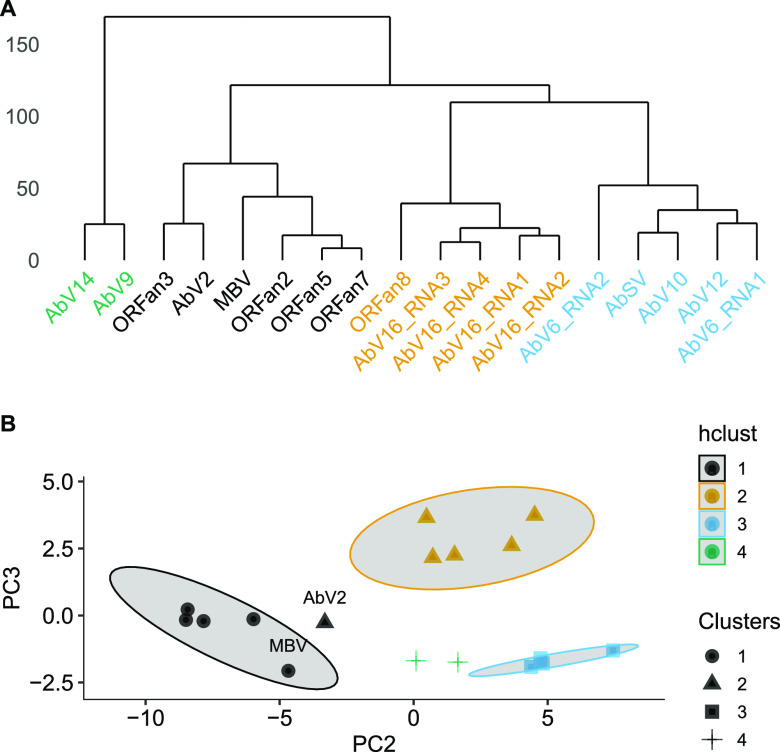
Viral abundance clustering. (A) Hierarchical cluster dendrogram computed on the Euclidean distance between RNA abundances using Ward's minimum variance method (45). (B) Principal component plot (PC2 versus PC3) of RNA abundances. Shapes indicate clusters identified with k-medoids clustering, and point colors indicate hierarchical clusters derived from panel A. The shaded area indicates the 85% confidence levels of each (hierarchical) cluster.

Viral RNAs were clustered on the basis of similar relative abundancies in the 89 samples using hierarchical and k-medoids clustering techniques (Fig. 1). Four clusters were identified by the k-medoids technique, determined by both gap statistics and within sum of squares (see Fig. S1 in the supplemental material). Both hierarchical and k-medoids methods produced clusters with very similar members (Table 1), with only AbV2 differing between the two. A silhouette plot showed there was very little difference in overall cluster scores between the two clustering methods for AbV2 (Fig. S2). Cluster 1 consists of ORFans 2, 3, 5, and 7 and, more loosely, AbV2; cluster 2 contains AbSV, AbV10, AbV12, AbV6 RNA1, and AbV6 RNA2; cluster 3 consists of the four components of AbV16 and ORFan8 (statistically confirming previously described associations [10, 23, 24]); and cluster 4 consists of AbV14 and AbV9. MBV was located in cluster 1 using hierarchical and k-medoids clustering techniques.

**TABLE 1:** Virus clustering as determined by hierarchical, k-medoid, principal component analysis, or pairwise correlation cluster methods^*a*^

RNA	Cluster method			
	Hierarchical	k-medoid	Principal component analysis	Pairwise correlation
ORFan2	1	1	1	1
ORFan5	1	1	1	1
ORFan7	1	1	1	1
ORFan3	1	1	1	1
MBV	1	1	1	Moderate clustering with 3
AbV2	1	2	Unclustered	1
AbSV	2	2	2	2
AbV12	2	2	2	2
AbV6 RNA1	2	2	2	2
AbV6 RNA2	2	2	2	2
AbV10	2	2	2	2
AbV16 RNA1	3	3	3	3
AbV16 RNA2	3	3	3	3
AbV16 RNA3	3	3	3	3
AbV16 RNA4	3	3	3	3
ORFan8	3	3	3	3
AbV14	4	4	4	4
AbV9	4	4	4	4

a Hierarchical clusters were determined by visual inspection of Fig. 1a.

10.1128/mBio.03470-20.4 FIG S1 Estimation of number of clusters for k-medoids clustering. (A) Gap statistics clusters estimated using the globalSEmax method (S. Dudoit and J. A. Fridlyand, Genome Biol 3:research0036.1, 2002, https://doi.org/10.1186/gb-2002-3-7-research0036). The vertical dashed blue line indicates the best number of clusters calculated from 500 bootstrap operations. (B) The number of clusters using the within sum of square method. The vertical dashed line indicates the elbow point of the graph identified by visual inspection. (C) Average silhouette scores for increasing numbers of clusters. The vertical dashed line indicates the best number of clusters found by visual inspection. Download FIG S1, PDF file, 0.1 MB. Copyright © 2021 Dobbs et al. 2021 Dobbs et al. https://creativecommons.org/licenses/by/4.0/ This is an open-access article distributed under the terms of the Creative Commons Attribution 4.0 International license.

10.1128/mBio.03470-20.5 FIG S2 Silhouette plots for k-medoids (A), hierarchical clustering (B), and pairwise correlation (C). The height of each column (the silhouette score) indicates the likelihood of the viral RNA belonging to the indicated cluster. The red horizontal dashed line indicates the mean silhouette score across all clusters. Download FIG S2, PDF file, 0.09 MB. Copyright © 2021 Dobbs et al. 2021 Dobbs et al. https://creativecommons.org/licenses/by/4.0/ This is an open-access article distributed under the terms of the Creative Commons Attribution 4.0 International license.

### Relationships within and between viral clusters: correlation and interactions.

**FIG 2:**
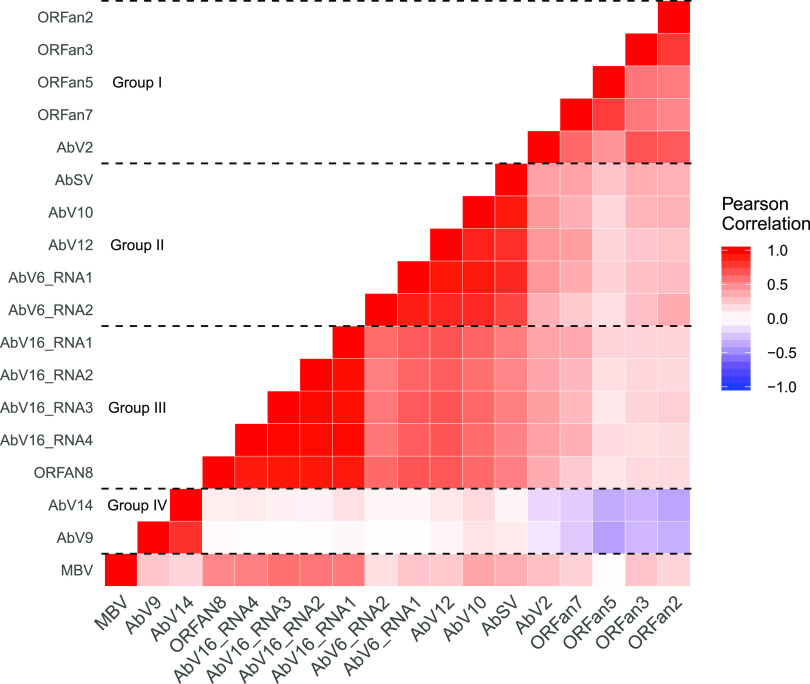
Pairwise correlation of abundance among nonhost RNAs. The RNA concentrations of 18 viral RNAs were measured by qPCR in 89 mushrooms from two separate cropping experiments and normalized using the host 18S gene to produce Δ*C_T_* values. The strengths of the correlations caused the RNAs to cluster into the 4 labeled groups of closely correlated expression.

Pairwise correlation of the abundances of all RNAs showed positive correlations within clusters and both positive and negative correlations between clusters (Fig. 2 and Fig. S3). Interaction between the groups was apparent; for instance, cluster 2 and cluster 3 had strong positive correlations among their member RNAs, while cluster 1 and cluster 4 member RNAs were negatively correlated (Fig. 2). MBV showed moderate correlation to cluster 3.

10.1128/mBio.03470-20.6 FIG S3 Viral abundance correlation groups. (A) Hierarchical clustering of abundance correlations. Principal component plots of clusters and grouping derived from Δ*C_T_* values for PC2 versus PC3 (B) and PC1 versus PC2 (C). Shapes indicate clusters identified with k-medoids clustering, and point color indicates group identified by pairwise correlation. Shaded areas indicate the 85% confidence levels of each group. Download FIG S3, PDF file, 0.2 MB. Copyright © 2021 Dobbs et al. 2021 Dobbs et al. https://creativecommons.org/licenses/by/4.0/ This is an open-access article distributed under the terms of the Creative Commons Attribution 4.0 International license.

### Population variance of viral clusters.

**FIG 3:**
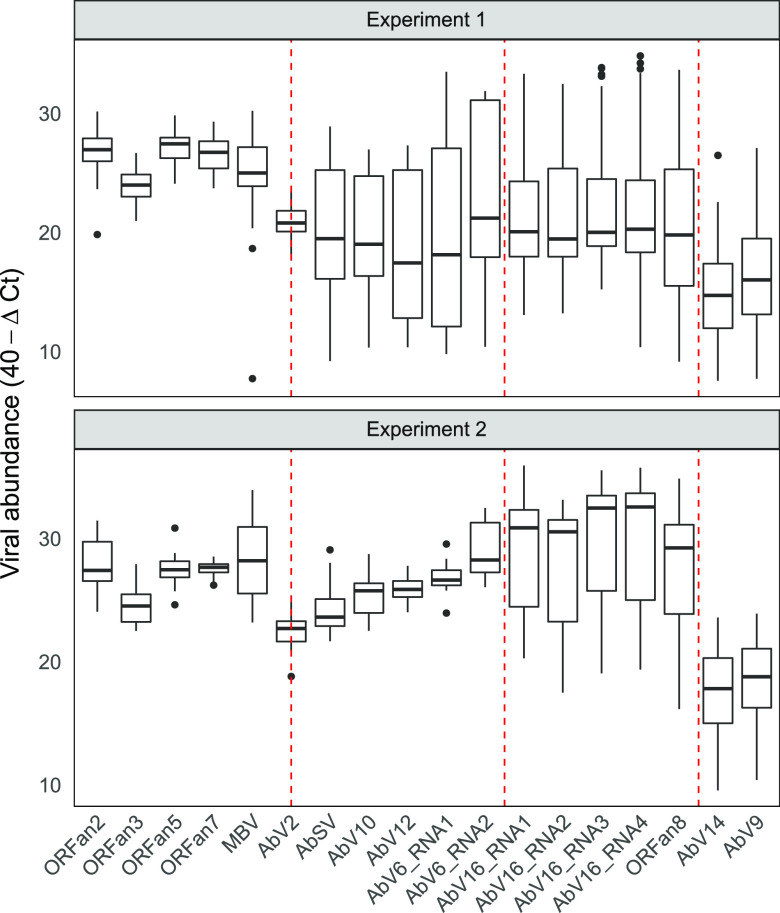
Distribution of viral population abundance in each experiment. The Δ*C_T_* values were transformed to 40−Δ*C_T_* values; therefore, higher values indicate higher abundance. RNA abundance box plots show the interquartile range between 25 and 75% of the data. Black bars are the sample medians. Whiskers extend to the largest (upper) and smallest (lower) values within 1.5× interquartile range from the hinge. Other values (outliers) are plotted individually. The vertical dashed red lines delineate the four viral clusters (cluster 1 to cluster 4). AbV2 is bisected due to its uncertain clustering.

Striking differences can be observed in the variance of cluster 2 between the two experiments (Fig. 3). These differences in variance were statistically significant (*P < *0.001). The large interquartile ranges for cluster 2 in experiment 1 contrast with much smaller interquartile ranges in experiment 2 (Fig. 3). No other clusters were significantly variant between the experiments (Fig. 3 and Fig. S4).

10.1128/mBio.03470-20.7 FIG S4 Mean cluster abundance for each experiment. The black points are the estimated marginal mean abundance for each cluster in each experiment calculated from the model (abundance ≈ experiment × cluster), and the purple bars represent the upper and lower 95% confidence intervals. Red arrows indicate, by degree of overlap (or lack thereof), the significance between contrasts, i.e., arrows that do not overlap have significantly different means. Download FIG S4, PDF file, 0.01 MB. Copyright © 2021 Dobbs et al. 2021 Dobbs et al. https://creativecommons.org/licenses/by/4.0/ This is an open-access article distributed under the terms of the Creative Commons Attribution 4.0 International license.

**FIG 4:**
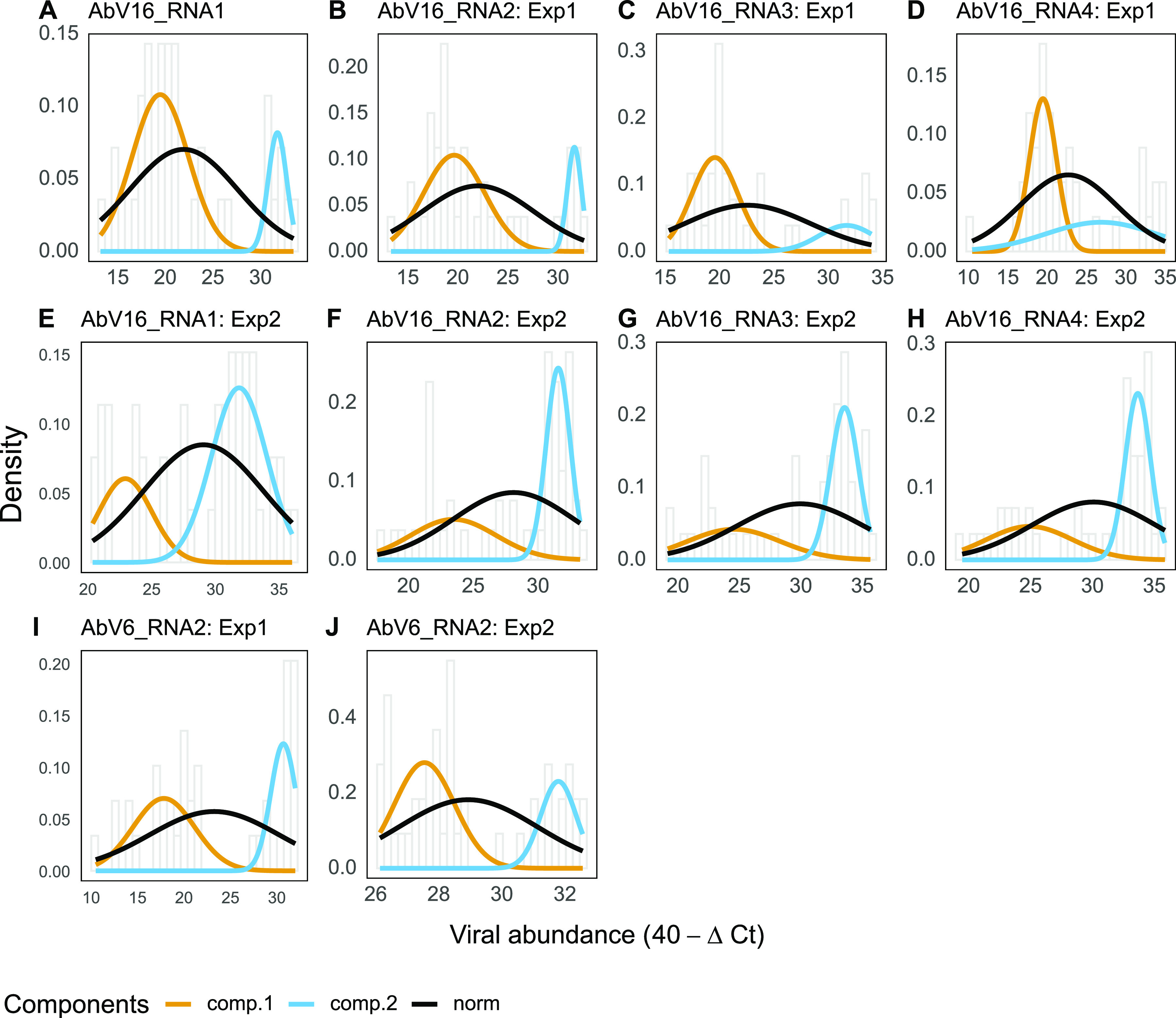
Best-fit normal models for viral RNA abundance in each experiment. Each graph shows a histogram of the viral abundance with bin width of 0.5 and normal (norm) and double normal (comp.1 and comp.2) curves. The double normal distribution model was fitted to the abundance of the named viral RNA (A to J) using expectation maximization, specifically by maximizing the conditional expected complete data log likelihood per viral abundance measurement (log likelihood and *P* values are given in Table S2 in the supplemental material).

Some of the viral RNAs appeared to be present in one of two populations, i.e., either at low or very high levels, with few intermediate values, as shown in the histograms of Fig. 4 and abundance density plots of Fig. S5. This was tested by looking for statistical evidence of bimodality by comparing the likelihood of fit to either a normal distribution or double normal distribution (indicating bimodality) using expectation maximization (Fig. 4). AbV16 RNAs 1 to 4 from cluster 3 and AbV6 RNA2 from cluster 2 fit a double normal distribution in both experiments and, therefore, were present in two distinct populations. The difference between the two peaks of abundance in AbV16 RNAs of cluster 3 was greater than 1,000-fold (>10 on the 40 − Δ*C_T_* scale, i.e., 2^10^) between the median values of high and low abundance. In addition, in experiment 1, AbVSV, AbV10, AbV12, and AbV6 RNA1 from cluster 2 had a better fit for a double normal distribution, whereas in experiment 2, a different set of additional viral RNAs, ORFans 2, 3, 7, and 8, fit better the double normal distribution (Fig. S6).

10.1128/mBio.03470-20.8 FIG S5 RNA abundance kernel density plots for AbV6 RNA2 and AbV16 RNA1. The *x* axis is viral abundance, and the area under the curve is the probability over a given range on the *x* axis. Download FIG S5, PDF file, 0.07 MB. Copyright © 2021 Dobbs et al. 2021 Dobbs et al. https://creativecommons.org/licenses/by/4.0/ This is an open-access article distributed under the terms of the Creative Commons Attribution 4.0 International license.

10.1128/mBio.03470-20.9 FIG S6 Best fit normal models for viral RNAs in each experiment. The normal (norm) and double normal (comp.1 and comp.2) distribution models were fitted to the viral RNA concentration data using expectation maximization, specifically by maximizing the conditional expected complete data log-likelihood per viral abundance measurement. Red boxes indicate a virus with a significantly higher fit to a double normal compared to normal abundance distribution (log likelihood and *P* values are given in Table S2). Download FIG S6, PDF file, 0.2 MB. Copyright © 2021 Dobbs et al. 2021 Dobbs et al. https://creativecommons.org/licenses/by/4.0/ This is an open-access article distributed under the terms of the Creative Commons Attribution 4.0 International license.

10.1128/mBio.03470-20.1 TABLE S1 A full list of the primers used for qRT-PCR. Download Table S1, DOCX file, 0.02 MB. Copyright © 2021 Dobbs et al. 2021 Dobbs et al. https://creativecommons.org/licenses/by/4.0/ This is an open-access article distributed under the terms of the Creative Commons Attribution 4.0 International license.

10.1128/mBio.03470-20.2 TABLE S2 Log likelihood and probability of double normal viral abundances. Download Table S2, DOCX file, 0.01 MB. Copyright © 2021 Dobbs et al. 2021 Dobbs et al. https://creativecommons.org/licenses/by/4.0/ This is an open-access article distributed under the terms of the Creative Commons Attribution 4.0 International license.

### Relationship between viral abundance and symptom expression.

**FIG 5:**
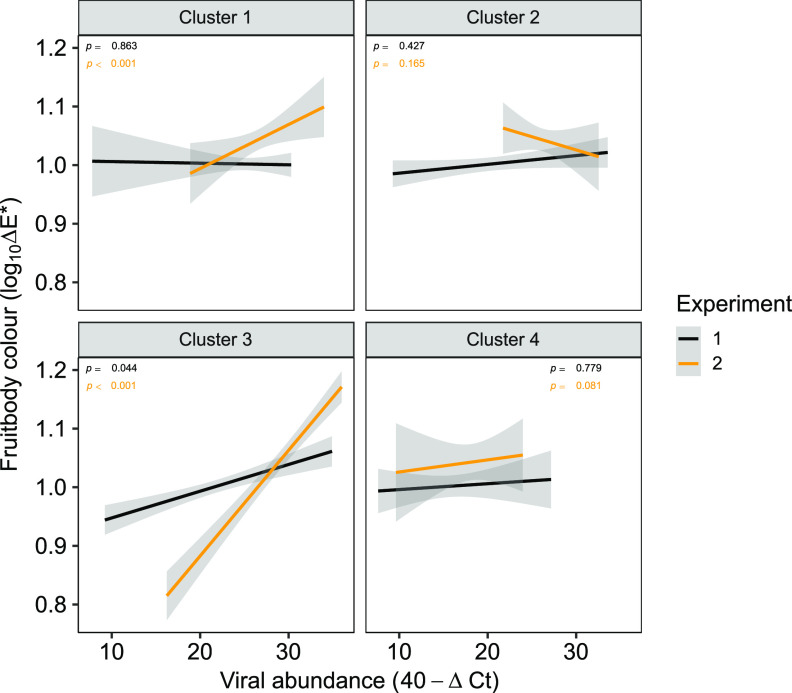
Cap color and viral abundance correlation in each viral cluster. High ΔE* values denote a brown color cap, and the Δ*C_T_* values have undergone a 40−Δ*C_T_* transformation so that higher 40−Δ*C_T_* values equate to high levels of viral RNA. *P* values are calculated from Pearson’s correlation coefficients. Trend lines were fitted using linear least-squares regression. Shaded areas around the lines indicate the 0.95 confidence interval.

**FIG 6:**
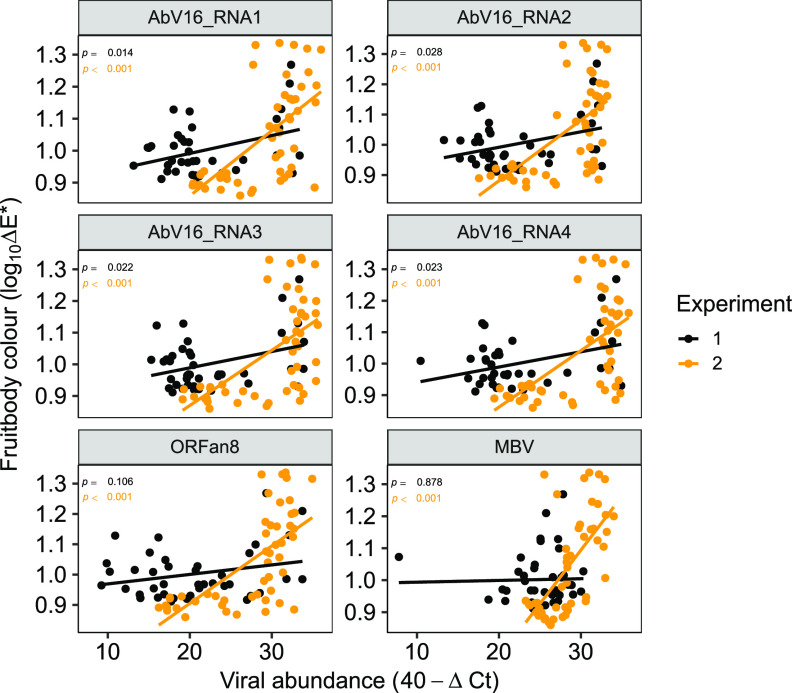
Cap color and viral abundance correlation. The abundance of the six presented viral RNAs significantly correlated (*P* ≤ 0.05) with cap color in either one or both experiments. The Δ*C_T_* values have undergone a 40−Δ*C_T_* transformation so that higher 40−Δ*C_T_* values equate to high levels of viral RNA. *P* values were calculated from Pearson’s correlation coefficients. Trend lines were fitted using linear least-squares regression.

Pearson’s pairwise correlations were calculated to identify possible correlations between symptom expression (mushroom cap color [log_10_ΔE]) and viral RNA abundance (40 − Δ*C_T_*) both collectively as clusters (Fig. 5) and as individual RNAs (Fig. 6). Significant correlations were established between the abundance of cluster 3 RNAs and cap color in both experiments 1 and 2, with the higher viral RNA abundance correlating with greater cap browning (Fig. 5). Cluster 1 abundance also significantly correlated with color but in experiment 2 only. Examination of correlations between individual RNA abundance and cap color enabled greater precision, and significant correlations were identified for the AbV16 components for both experiments and for ORFan8 and MBV in experiment 2 (Fig. 6 and Table S3).

10.1128/mBio.03470-20.3 TABLE S3 Correlation between cap color and RNA abundance. Download Table S3, DOCX file, 0.01 MB. Copyright © 2021 Dobbs et al. 2021 Dobbs et al. https://creativecommons.org/licenses/by/4.0/ This is an open-access article distributed under the terms of the Creative Commons Attribution 4.0 International license.

### Comparison of AbV16 viral levels in fruitbodies and underlying mycelium.

**FIG 7:**
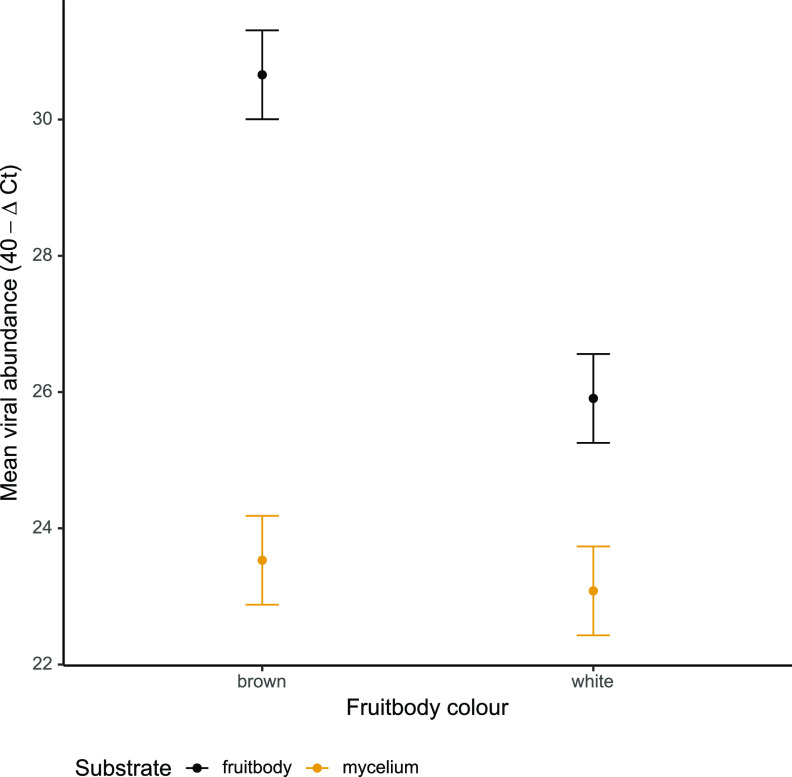
Mean abundance of AbV16 (RNA1 and RNA2) in 8 brown and 8 white mushrooms and from the mycelium directly below the sampled mushrooms estimated from the linear model. Error bars show the standard errors.

RNA levels of AbV16 RNA 1 and RNA 2 were compared between 16 fruitbodies (8 symptomatic brown and 8 white mushrooms) and the underlying mycelium directly below these 16 mushrooms, a subset of the total data. By blocking the variance due to the difference between the RNA types, a clear difference was identified in AbV16 abundance between brown and white fruitbodies (*t* = 5.149, df = 43, *P < *0.001), but no difference was found in AbV16 abundance between the mycelia below brown and white fruitbodies (*t* = 0.488, df = 43, *P = *0.628) (Fig. 7).

## DISCUSSION

*Agaricus bisporus* mycelium (strain A15) harboring a relatively low number of viral RNAs was infected with additional viral RNAs from a small inoculum of MVX-4569 *A. bisporus* mycelium. The viral RNAs moved and replicated throughout the A15 mycelium, including the mycelium of the casing layer from where fruitbodies developed following environmental manipulation. Eighteen viral RNAs representing nine viruses were detected in the cultures, and their abundances were analyzed to elucidate their relationships and how they interact. The nine viruses in the cultures represent nine distinct RNA-dependent RNA polymerase (RdRp) enzymes with the potential for competing for resources required for viral replication. However, these RNAs could be grouped into four clusters using unsupervised statistical measures of their abundances. The grouping of AbV2 and MBV into cluster 1 was less strong. The identification of clusters suggests that virus levels had reached equilibrium in the interval between infection and fruitbody sample harvest (20 days).

The abundance of the four components of AbV16 correlated with the disease symptom, cap browning. The levels of AbV16 RNAs also interacted positively with viral levels of a different cluster, cluster 2, by pairwise correlation. In addition, the components of AbV16 and AbV6 RNA2 (cluster 2) exhibited two distinct populations of RNA levels, low and very high, orders of magnitude apart. We propose that the disease phenotype is caused by interaction between these clusters leading to the step-jump in viral RNA levels. However, we acknowledge the possibility of the inverse, that the changes in viral abundance are a response to the cellular pathology. There appears to be a fitness cost to the step-jump in RNA levels, as AbV16 abundance can crash in culture after exhibiting the disease culture (23). High levels of AbV16 have been found early during fruitbody development, and thereafter there is no evidence of transition between the two viral replicative states; levels remain either low or very high (23). Here, we show that before fruitbody formation, AbV16 levels were low in the mycelium. At the point of environmental stimulation for differentiation to form fruitbodies, 15% of the developing fruitbodies underwent an abrupt shift to 1,000 times higher levels of AbV16, and for the remaining fruitbodies the levels stayed low.

The clusters represent evidence of synergism and viral coreplication, probably resulting from different mechanisms. Cluster 3 RNAs have previously been proposed as a single virus, AbV16 and the tightly associated ORFan8 (10), and therefore is replicated by the single RdRp of the cluster. Cluster 1 (ORFans 2, 3, 5, and 7 and, more loosely, AbV2 and MBV), cluster 2 (AbSV, AbV10, AbV12, AbV6 RNA1, and AbV6 RNA2), and cluster 4 (AbV9 and AbV14) consist of phylogenetically diverse viruses with multiple RdRps, which, therefore, are unlikely to be replicated by a single replicase. The mechanisms by which these RNAs cluster, implying coordinated replication, are not fully understood but may involve complementary suppression or saturation of host antiviral defenses or forms of molecular interactions that are similar to the collective infectious units that enhance comovement and replication (18, 21, 28, 29). In *C. parasitica*, the suppression of the host antiviral silencing mechanism by the hypovirus CHV4 enabled the stable coinfection of the reovirus MyRV2 (4).

The replication of all positive-strand RNA viruses is generally associated with cellular membranes that are heavily modified or reorganized to act as efficient platforms for genome amplification (30). Major membrane modification is consistent with the cause of the browning reaction (by pathology or mechanical damage), i.e., the oxidation of phenols by tyrosinase, which are separated into different compartments by membranes (31). Thus, membrane modification and reorganization plausibly lead to leakiness, in turn promoting the reaction and the formation of the brown-colored products. The exponentially higher replicative capacity of the step-jump in viral levels may involve additional cellular factors, such as the centrosome-microtubule-binding domain gene, which has been reported to be upregulated in *A. bisporus* with high viral levels, or structural proteins encoded by the viruses, possibly acting as scaffolds for viral replication complexes (23). The two viral RNAs with known structural protein domains are AbV6 RNA2 and MBV, each of which encodes capsid-like domains (10, 32). RNA silencing is the primary antiviral defense mechanism in fungi; however, it is unknown whether its suppression alone could account for the 1,000-fold increase in viral levels or whether an additional mechanism of high-throughput viral replication is involved. The step-jump in viral RNA replication appears to be generic and not sequence specific, as a number of additional RNAs underwent this change in one experiment only.

Correlations both positive (clusters 2 and 3) and negative (clusters 1 and 4) were identified between individual clusters (Fig. 2). A lack of correlations also was observed between cluster 1 and clusters 2 and 3 and between cluster 4 and clusters 2 and 3, indicating neutral or no interactions. The mechanism for the antagonism between clusters 1 and 4 could be negative dominance, whereby defective interfering RNAs (such as the ORFans of cluster 1) interfere with viral replication in a cell by faster but defective replication (21, 33). RNA silencing can contribute to the production of defective interfering RNA (20). Cluster 1 consists of AbV2, MBV, and ORFans 2, 3, 5, and 7. AbV2 has been described as hypovirus-like based on its 28% sequence identity at the protein level to *Cryphonectria hypovirus 2* (10). There is evidence that ORFan3 is subgenomic to AbV2, as they are joined in some sequence assemblies (10). Cluster 1 included many RNAs previously described as ubiquitous and asymptomatic, consistent with the observation that persistent viruses minimize the negative effects of parasitizing their host or confer an advantage on the host in compensation, the trade-off hypothesis (34, 35). An alternative mechanism for the antagonism could be mutual exclusion, i.e., that viruses of each cluster are spatially separated in different cells (18). This hypothesis was not tested in these experiments, as the samples consisted of whole fruitbodies.

MBV did not consistently group with the clusters. The hierarchical and k-medoid clustering methods placed MBV with cluster 1 (Fig. 1), but it falls outside clusters from principal component analysis (see Fig. S3 in the supplemental material), and pairwise correlation showed MBV correlating with RNAs of cluster 3 (Fig. 2). Unlike AbV16, MBV did not have a bimodal distribution of abundance, although its abundance correlated with cap browning in experiment 2. MBV, a particulate virus, also has been found as a coinfection of AbV1, where it appears to exploit incidents of high viral replication (36).

There appears to be a stochastic nature to the viral interactions and the development of pathogenesis. For instance, (i) brown symptomatic fruitbodies were distributed apparently randomly in beds of growing white mushrooms, (ii) large differences were identified in variance of cluster 2 RNAs between experiments 1 and 2 (Fig. 3A), and (iii) the viruses with double normal distributions differed between experiments 1 and 2 (Fig. 4). That stochastic factors could play a role in the complexity of the multifactorial and dynamic state of a large number of viral RNAs is perhaps not surprising, an otherwise jostling equilibrium tipped over the edge by the microenvironment. The RNAs that consistently demonstrated the exponential step-jump comprise two multipartite viruses, AbV16 and the capsid-encoding AbV6 RNA2, and the stochastic event may result from a combination of these RNAs at the same cellular location at a specific phase in the cell (duplication) cycle during early fruitbody formation. In mycelial cells, AbV16 and AbV6 RNA2 have been shown to be separately located using fluorescence *in situ* hybridization (37).

Rather than competing for the cells’ resources, the numerous viruses interacted synergistically as groups, as shown by their similar relative abundances. Further interactions between groups were identified as synergistic, antagonistic, and neutral. This statistical and modeling study has revealed simultaneous viral interactions in the same fungal culture. The study did not examine mechanisms of interaction, but the data are consistent with previously described mechanisms, such as suppression of antiviral defenses, defective interfering RNA, spatial separation, and replication by a single RdRp. It is unknown whether these mechanisms can collectively account for the 1,000-fold increase in AbV16 and AbV6 RNA2 levels. Host gene expression changes, viral translocation, and effects of environmental stresses will be subject to further research. Multiple viral infections are likely to be prevalent in nature, although they may be undetected, as they are asymptomatic. However, the random expression of severe symptoms may be a factor in the uncertainties associated with biological control agents, such as fungi and viruses, in controlling fungal diseases.

## MATERIALS AND METHODS

### Biological material.

Two sources of *Agaricus bisporus* were used, (i) commercial spawn of Sylvan strain A15 (Sylvan Inc., Kittanning, PA, USA) and (ii) the virus-infected strain 4569 (MVX-4569), which was isolated and cultured from a diseased fruitbody originally collected in 2005 from a commercial mushroom farm growing strain A15 (23) and maintained in the laboratory as mycelial subcultures per Grogan et al. (24). Experimental inoculum of MVX-4569 was produced as per Fleming-Archibald et al. (38). The inoculum was stored at 4°C for 5 months between the two experiments.

### Experimental conditions.

Two experiments were carried out under the same growth conditions but at different scales: experiment 1, large scale, where mushrooms were grown on a metal shelf system (0.24 m by 0.24 m by 4 m), reflecting common industry practice for growing mushrooms, and experiment 2, small scale, where mushrooms were grown in three polypropylene crates (0.6 m by 0.4 m by 0.24 m).

In both experiments, pasteurized mushroom compost, known commercially as phase II compost, was inoculated with 0.5% (wt/wt) commercial A15 spawn. For experiment 1, the metal shelf was filled with 160 kg of inoculated phase II mushroom compost. For experiment 2, three polypropylene crates were each filled with 15 kg of inoculated phase II mushroom compost. For each experiment after the filling process, the mycelium was allowed to colonize the compost for 17 days in a controlled environment at 25°C, >6,000 ppm CO_2_, and 90 to 95% relative humidity per standard mushroom industry practice.

The fully colonized compost was then inoculated with MVX-4569-infected compost fragments by incorporating them into the surface of the A15 colonized compost. In experiment 1, 2 g of MVX-4569-infected compost fragments was incorporated into the first 0.5-m length of compost in the trough (approximately 20 kg). In experiment 2, 1.5 g was incorporated uniformly into the surface layer of the colonized compost in the polypropylene crates (15 kg). The effective rate of inoculation in both cases was 0.01%, as described by Fleming-Archibald et al. (38).

The compost for both experiments was then covered with 55 mm of casing soil (Harte Peat; Clones Co., Monaghan, Ireland), incubated for a further 7 days under the same conditions as detailed above. Mushroom fruitbody production was initiated by an environmental manipulation, reduction of CO_2_ levels to 1,200 ppm and temperature to 18°C (38). Mushroom fruitbodies from the first flush were harvested for experimental use 20 days after casing application. In both experiments, the inoculation with MVX-4569 resulted in the production of symptomatic brown-colored mushrooms (10 to 15%) randomly distributed among white asymptomatic mushrooms largely in the zone where inoculation had taken place.

### Fruitbody and compost sampling.

In experiment 1, 40 mushroom fruitbodies were harvested from the growing shelf; 10 of these were visibly the brownest in color, and three white fruitbodies near each brown mushroom were also selected and harvested. For experiment 2, 49 fruitbody samples were harvested with various degrees of color symptoms, and 16 compost samples were taken directly below specific fruitbodies, 8 from below fruitbodies displaying the brown symptom and the remaining 8 from below asymptomatic fruitbodies, white in color. To sample the compost, the fruitbody was harvested, the casing layer was carefully removed, and approximately 5 g of the compost below was collected. All samples were harvested by hand. After color measurement, the samples were freeze-dried for approximately 24 h until completely dry and stored at −20°C.

### Mushroom cap color determination.

Mushroom cap color was measured using a CR-410 Chromameter (Konica Minolta, Basildon, UK) as three color parameters, L*, b*, and a*, which were then combined as a single parameter, ΔE (39).

### RNA extraction from fruitbodies.

RNA was extracted from mushroom fruitbodies using a modified version of our previously published methodology (10). Briefly, 80 mg dry weight of mushroom fruitbody was mixed with 4× (wt/vol) lysis buffer (STE, 1% SDS) and 4× (wt/vol) 5:1 phenol-chloroform (pH 4.5) (Fisher Scientific, Loughborough, UK), and cells were lysed using bead beating (Retsch, Hann, Germany). RNA was precipitated by adding 0.1 volumes of 3 M sodium acetate, pH 5.2, and 2 volumes of absolute ethanol. The RNA was then cleaned by running through 10% polyvinylpolypyrrolidone (PVPP) spin columns. If the flowthrough retained a strong color, it was applied to a further PVPP column and the procedure repeated. RNA quality and quantity were measured using a NanoDrop 1000 spectrophotometer (Thermo Scientific, Loughborough, UK) and the samples stored at −80°C.

### RNA extraction from compost.

RNA was extracted from compost using a methodology similar to that for fruitbodies. A volume of 75 mg of freeze-dried compost was mixed with 1 ml TRIzol (Sigma-Aldrich, Haverhill, UK) and cells lysed by bead beating. RNA was then recovered by binding to 50 μl of silica gel in suspension. The silica was pelleted by pulse spinning on a microcentrifuge for 10 s and RNA cleaned by sequentially washing in (i) 1 ml wash buffer (100 ml 0.1 M Tris-HCl, pH 6.4, and 120 g guanidine thiocyanate), (ii) 1 ml wash buffer, (iii) 1 ml 70% ethanol, (iv) 1 ml 70% ethanol, and (v) 1 ml acetone with pulse spinning and discarding of the supernatant at each step. RNA was resuspended in 50 μl RNase-free water and stored at −80°C.

### DNase treatment and reverse transcription.

The resulting nucleic acid was treated with RNase-free DNase 1 (Fisher Scientific, Loughborough, UK) by adding 1 μl DNase 1 (1 U/μl), 1 μl 10× reaction buffer, nucleic acid at 1,000 ng, and nuclease-free water up to 10 μl in a nuclease-free 0.2-ml PCR tube (Fisher Scientific, Loughborough, UK) on ice. These were incubated at 37°C for 60 min. To inactivate the DNase, 1 μl 50 mM EDTA was added and incubated at 65°C for 10 min. The resulting RNA was reverse transcribed to cDNA by adding to a nuclease-free 0.2-ml PCR tube on ice, 1.2 μl random hexamer primer (Thermo Scientific, Loughborough, UK), 2 μl deoxynucleotide triphosphates (5 mM each) (Fisher Bioreagents), 1 μl RNA, and 8.8 μl nuclease-free water. This was incubated at 65°C for 5 min. It was then placed on ice for at least 1 min. Four microliters of 5× first-strand buffer, 1 μl 0.1 M dithiothreitol, 1 μl nuclease-free water, and 1 μl reverse transcriptase (Superscript III; 200 U/μl) (Invitrogen, Loughborough, UK) was added, and the tubes were incubated at 25°C for 5 min, 50°C for 60 min, and 70°C for 15 min.

### qPCR.

The qPCR assays were performed in duplicate using reaction mixtures containing 7.5 μl SYBR green master mix (Applied Biosystems, Loughborough, UK), 1 μl diluted cDNA, 15 pM forward and reverse primers, and nuclease-free water to 15 μl. In addition, nontemplate water control was included for each primer set on every plate. Detection and quantification were achieved with an Applied Biosystems 7500 real-time PCR system (Applied Biosystems, Loughborough, UK). The following amplification cycles were used: 2 min at 50°C, 10 min at 95°C, and 40 cycles of 15 s at 95°C and 60 s at 60°C. The cycle threshold (*C_T_*) values for the viral RNAs were normalized against the 18S gene to determine the Δ*C_T_* values used for analysis.

### Statistical analysis.

Several statistical approaches were taken to identify novel associations between viruses and, by inference, their interactions. All statistical analyses were carried out in R 3.4.0 (40). Two unsupervised clustering methods, hierarchical and k-medoids (41), and principal component analysis (PCA) were applied to the abundance data of all 89 fruitbody samples to identify viral clusters. Properties of viral clusters were investigated using further techniques. Pearson’s pairwise correlations were used to identify (i) positive and negative interactions between viruses and their clusters, (ii) the relationship between viral abundance and fruitbody color, and (iii) the relationship between RNA abundance in fruitbodies and the underlying mycelium. Differences in the variance of abundance between (i) clusters, (ii) experiments, and (iii) their interaction were tested by fitting a linear model to the absolute deviance from the median value of each virus per experiment. Significance of each term in the model was calculated with analysis of variance (ANOVA). Estimated marginal means and *post hoc* comparison test were calculated with emmeans (42) with comparison *P* values adjusted for multiple testing using the Tukey method (43). To determine whether the sample abundances of each RNA fit a single or double normal distribution, single and double normal models were fit to the data using expectation maximization implemented in the mixtools package (44); significance was calculated using bootstrapping by producing 10,000 iterations of the likelihood ratio statistic for testing the null hypothesis-single normal fit versus the alternative hypothesis-double normal fit with significance for rejecting the null taken at the 0.05 level.

To examine any differences in the abundances of AbV16 RNA1 and RNA2 between underlying mycelia and fruitbodies, diseased and asymptomatic, data were fit to a linear model, and analysis of variance was used to identify differences between (i) the RNAs, (ii) fruitbody color, (iii) tissue type (fruitbody or mycelium), and (iv) the interactions (RNA-color-tissue) while controlling for paired sample (fruitbody and underlying mycelium). The significance of each term in the model was calculated with ANOVA. Estimated marginal means and *post hoc* comparison test were calculated with emmeans (42) with contrast *P* values controlled for familywise error rate using the Tukey method (43).

## References

[1] Karyeija RF, Kreuze JF, Gibson RW, Valkonen JPT. 2000. Synergistic interactions of a potyvirus and a phloem-limited crinivirus in sweet potato plants. Virology 269:26–36. doi:10.1006/viro.1999.0169. 10725195

[2] Rentería-Canett I, Xoconostle-Cázares B, Ruiz-Medrano R, Rivera-Bustamante RF. 2011. Geminivirus mixed infection on pepper plants: synergistic interaction between PHYVV and PepGMV. Virol J 8:104. doi:10.1186/1743-422X-8-104. 21385390 PMC3061938

[3] Harper SJ, Cowell SJ, Dawson WO. 2015. Finding balance: virus populations reach equilibrium during the infection process. Virology 485:205–212. doi:10.1016/j.virol.2015.07.017. 26291064

[4] Aulia A, Andika IB, Kondo H, Hillman BI, Suzuki N. 2019. A symptomless hypovirus, CHV4, facilitates stable infection of the chestnut blight fungus by a coinfecting reovirus likely through suppression of antiviral RNA silencing. Virology 533:99–107. doi:10.1016/j.virol.2019.05.004. 31146252

[5] Maliogka VI, Martelli GP, Fuchs M, Katis NI. 2015. Control of viruses infecting grapevine. Adv Virus Res 91:175–227. doi:10.1016/bs.aivir.2014.11.002. 25591880

[6] Nerva L, Turina M, Zanzotto A, Gardiman M, Gaiotti F, Gambino G, Chitarra W. 2019. Isolation, molecular characterization and virome analysis of culturable wood fungal endophytes in esca symptomatic and asymptomatic grapevine plants. Environ Microbiol 21:2886–2904. doi:10.1111/1462-2920.14651. 31081982

[7] Zhu JZ, Zhu HJ, Da Gao B, Zhou Q, Zhong J. 2018. Diverse, novel mycoviruses from the virome of a hypovirulent sclerotium rolfsii strain. Front Plant Sci 9:1738. doi:10.3389/fpls.2018.01738. 30542362 PMC6277794

[8] Herrero N, Dueñas E, Quesada-Moraga E, Zabalgogeazcoa I. 2012. Prevalence and diversity of viruses in the entomopathogenic fungus Beauveria bassiana. Appl Environ Microbiol 78:8523–8530. doi:10.1128/AEM.01954-12. 23001673 PMC3502908

[9] Osaki H, Sasaki A, Nomiyama K, Tomioka K. 2016. Multiple virus infection in a single strain of Fusarium poae shown by deep sequencing. Virus Genes 52:835–847. doi:10.1007/s11262-016-1379-x. 27550368

[10] Deakin G, Dobbs E, Bennett JM, Jones IM, Grogan HM, Burton KS. 2017. Multiple viral infections in Agaricus bisporus—characterisation of 18 unique RNA viruses and 8 ORFans identified by deep sequencing. Sci Rep 7:2469. doi:10.1038/s41598-017-01592-9. 28550284 PMC5446422

[11] Gilbert KB, Holcomb EE, Allscheid RL, Carrington JC. 2019. Hiding in plain sight: new virus genomes discovered via a systematic analysis of fungal public transcriptomes. PLoS One 14:e0219207. doi:10.1371/journal.pone.0219207. 31339899 PMC6655640

[12] Hillman BI, Shapira R, Nuss DL, . 1990. Hypovirulence-associated suppression of host functions in Cryphonectria parasitica can be partially relieved by high light intensity. Phytopathology 80:950–956. doi:10.1094/Phyto-80-950.

[13] Hull R. 1996. Molecular biology of rice tungro viruses. Annu Rev Phytopathol 34:275–297. doi:10.1146/annurev.phyto.34.1.275. 15012544

[14] Mahuku G, Lockhart BE, Wanjala B, Jones MW, Kimunye JN, Stewart LR, Cassone BJ, Sevgan S, Nyasani JO, Kusia E, Kumar PL, Niblett CL, Kiggundu A, Asea G, Pappu HR, Wangai A, Prasanna BM, Redinbaugh MG. 2015. Maize lethal necrosis (MLN), an emerging threat to maize-based food security in sub-Saharan Africa. Phytopathology 105:956–965. doi:10.1094/PHYTO-12-14-0367-FI. 25822185

[15] Pio-Ribeiro G, Wyatt SD, Kuhn CW, . 1978. Cowpea stunt: a disease caused by a synergistic interaction of two viruses. Phytopathology 68:1260–1265. doi:10.1094/Phyto-68-1260.

[16] Sicard A, Yvon M, Timchenko T, Gronenborn B, Michalakis Y, Gutierrez S, Blanc S. 2013. Gene copy number is differentially regulated in a multipartite virus. Nat Commun 4:2248. doi:10.1038/ncomms3248. 23912259

[17] DaPalma T, Doonan BP, Trager NM, Kasman LM. 2010. A systematic approach to virus-virus interactions. Virus Res 149:1–9. doi:10.1016/j.virusres.2010.01.002. 20093154 PMC7172858

[18] Syller J. 2012. Facilitative and antagonistic interactions between plant viruses in mixed infections. Mol Plant Pathol 13:204–216. doi:10.1111/j.1364-3703.2011.00734.x. 21726401 PMC6638836

[19] Elena SF, Bernet GP, Carrasco JL. 2014. The games plant viruses play. Curr Opin Virol 8:62–67. doi:10.1016/j.coviro.2014.07.003. 25062019

[20] Nuss DL. 2011. Mycoviruses, RNA silencing, and viral RNA recombination. Adv Virus Res 80:25–48. doi:10.1016/B978-0-12-385987-7.00002-6. 21762820 PMC3313461

[21] Sanjuán R. 2017. Collective infectious units in viruses internet. Trends Microbiol Elsevier Curr Trends 25:402–412. doi:10.1016/j.tim.2017.02.003. PMC5837019 28262512

[22] Van Der Lende TR, Duitman EH, Gunnewijk MGW, Yu L, Wessels JGH. 1996. Functional analysis of dsRNAs (L1, L3, L5, and M2) associated with isometric 34-nm virions of Agaricus bisporus (white button mushroom). Virology 217:88–96. doi:10.1006/viro.1996.0096. 8599239

[23] Eastwood D, Green J, Grogan H, Burton K. 2015. Viral agents causing brown cap mushroom disease of Agaricus bisporus. Appl Environ Microbiol 81:7125–7134. doi:10.1128/AEM.01093-15. 26253676 PMC4579443

[24] Grogan HM, Adie BAT, Gaze RH, Challen MP, Mills PR. 2003. Double-stranded RNA elements associated with the MVX disease of Agaricus bisporus. Mycol Res 107:147–154. doi:10.1017/s0953756203007202. 12747325

[25] Revill P. 2008. Barnaviruses, p 286–288. *In* Mahy BWJ, van Regenmortel MHV (ed), Encyclopedia of virology, 3rd ed. Elsevier Academic Press, Amsterdam, The Netherlands.

[26] Nibert ML, Manny AR, Debat HJ, Firth AE, Bertini L, Caruso C. 2018. A barnavirus sequence mined from a transcriptome of the Antarctic pearlwort Colobanthus quitensis. Arch Virol 163:1921–1926. doi:10.1007/s00705-018-3794-x. 29516246 PMC5999160

[27] Starr EP, Nuccio EE, Pett-Ridge J, Banfield JF, Firestone MK. 2019. Metatranscriptomic reconstruction reveals RNA viruses with the potential to shape carbon cycling in soil. bioRxiv https://www.biorxiv.org/content/10.1101/597468v1.abstract. 10.1073/pnas.1908291116 PMC6926006 31772013

[28] Elena SF, Fraile A, García-Arenal F. 2014. Evolution and emergence of plant viruses. Adv Virus Res 88:161–191. doi:10.1016/B978-0-12-800098-4.00003-9. 24373312

[29] Dall’ara M. 2018. RNA/RNA interactions involved in the regulation of Benyviridae viral cycle. PhD thesis. Université de Strasbourg, Strasbourg, France. http://www.theses.fr/2018STRAJ019/document.

[30] Kallio K, Hellström K, Jokitalo E, Ahola T. 2016. RNA replication and membrane modification require the same functions of alphavirus nonstructural proteins. J Virol 90:1687–1692. doi:10.1128/JVI.02484-15. 26581991 PMC4719637

[31] Burton KS. 1988. The effects of pre- and post-harvest development on mushroom tyrosinase. J Hortic Sci 63:255–260. doi:10.1080/14620316.1988.11515856.

[32] Goodin MM. 1992. Encapsidation of the La France disease-specific double-stranded RNAs in 36-nm isometric viruslike particles. Phytopathology 82:285. doi:10.1094/Phyto-82-285.

[33] Marriott AC, Dimmock NJ. 2010. Defective interfering viruses and their potential as antiviral agents. Rev Med Virol 20:51–62. doi:10.1002/rmv.641. 20041441

[34] Alizon S, Hurford A, Mideo N, Van Baalen M. 2009. Virulence evolution and the trade-off hypothesis: history, current state of affairs and the future. J Evol Biol 22:245–259. doi:10.1111/j.1420-9101.2008.01658.x. 19196383

[35] Márquez LM, Roossinck MJ. 2012. Do persistent RNA viruses fit the trade-off hypothesis of virulence evolution? Curr Opin Virol 2:556–560. doi:10.1016/j.coviro.2012.06.010. 22819020

[36] Romaine CP, Schlagnhaufer B. 1995. PCR analysis of the viral complex associated with La France disease of Agaricus bisporus. Appl Environ Microbiol 61:2322–2325. doi:10.1128/AEM.61.6.2322-2325.1995. 7793952 PMC167503

[37] O'Connor E, Coates CJ, Eastwood DC, Fitzpatrick DA, Grogan H. 2020. FISHing in fungi: visualisation of mushroom virus X in the mycelium of Agaricus bisporus by fluorescence in situ hybridisation. J Microbiol Methods 173:105913. doi:10.1016/j.mimet.2020.105913. 32275924

[38] Fleming-Archibald C, Ruggiero A, Grogan HM. 2015. Brown mushroom symptom expression following infection of an Agaricus bisporus crop with MVX associated dsRNAs. Fungal Biol 119:1237–1245. doi:10.1016/j.funbio.2015.09.004. 26615746

[39] Sharma G. 2017. Digital color imaging handbook. CRC Press, Boca Raton, FL.

[40] R Core Development Team. 2013. A language and environment for statistical computing. R Core Team, Vienna, Austria. https://www.r-project.org/

[41] Maechler M, Rousseeuw P, Struyf A, Hubert M, Hornik K. 2019. cluster: cluster analysis basics and extensions. R package version 2.1.0. https://cran.r-project.org/package=cluster.

[42] Lenth R. 2018. Emmeans: estimated marginal means, aka least-squares means. https://cran.r-project.org/package=emmeans.

[43] Tukey JW. 1949. Comparing individual means in the analysis of variance Int Biometric Soc 5:99–114. doi:10.2307/3001913. 18151955

[44] Benaglia T, Chauveau D, Hunter DR, Young D. 2009. mixtools: an R package for analyzing finite mixture models. J Stat Softw 32:1–29. http://www.jstatsoft.org/v32/i06/.

[45] Ward DH. 1963. Comparison of different systems of exponentially weighted prediction. J R Stat Soc Ser D Stat 13:173–191. doi:10.2307/2986810.

