# Supplementary figures and images for: Viral Interactions and Pathogenesis during Multiple Viral Infections in Agaricus bisporus
Source: mBio. 2021 Feb 9;12(1):e03470-20. doi: 10.1128/mBio.03470-20 (PMC8545118; doi:10.1128/mBio.03470-20)

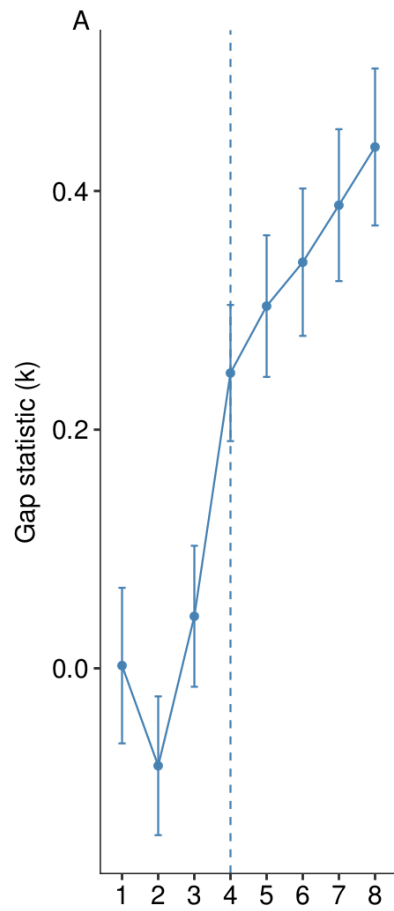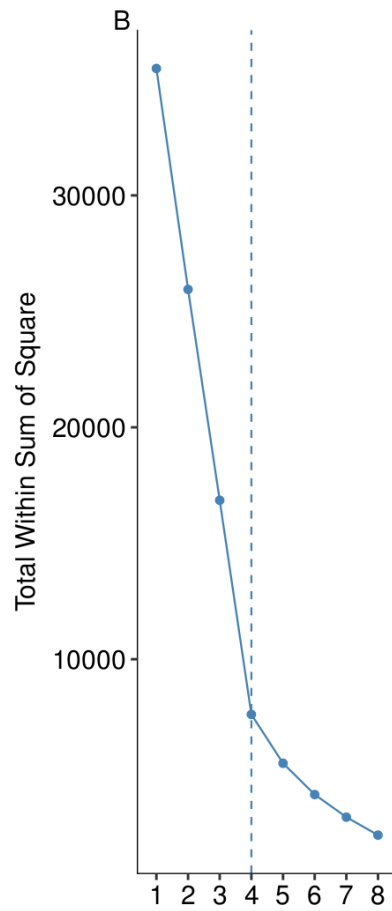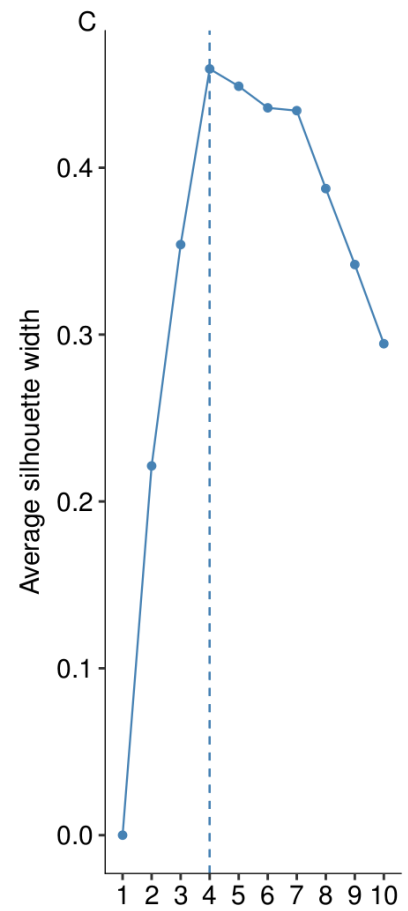

Number of clusters

Supplement: FIG S1 [file mbio.03470-20-sf001.pdf]

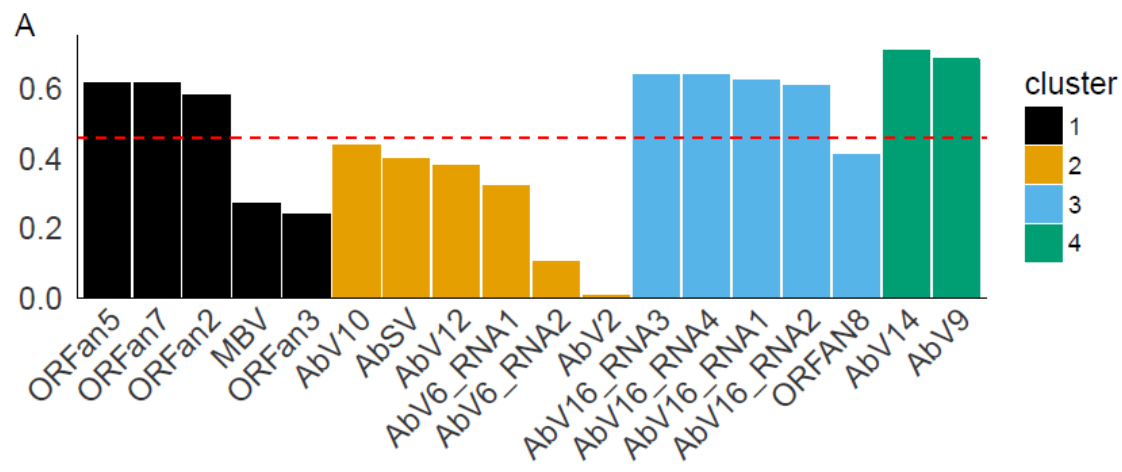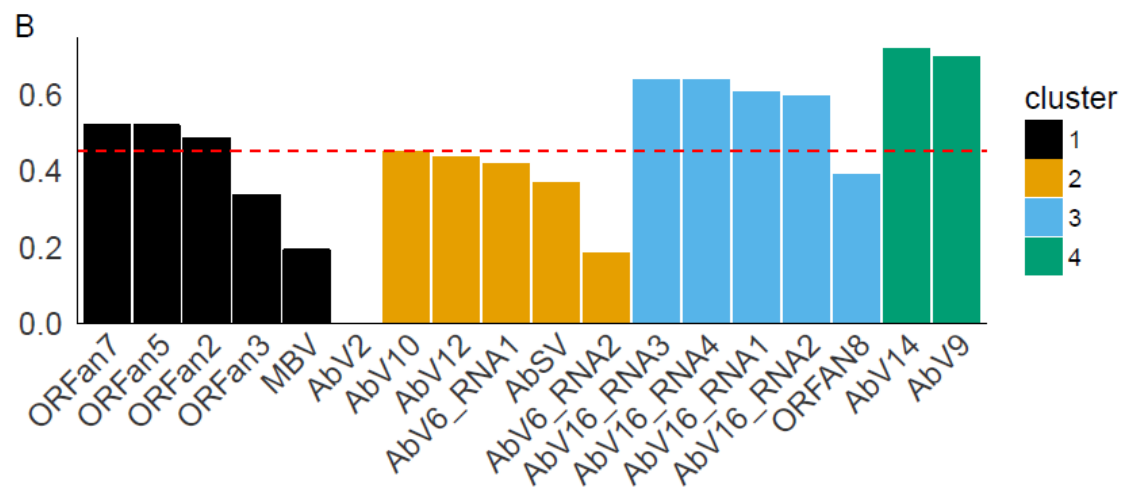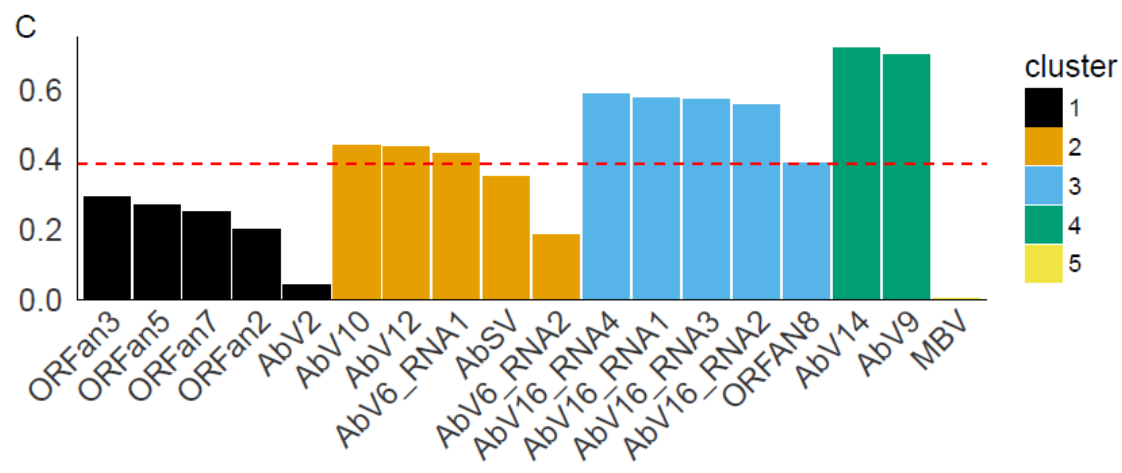

Supplement: FIG S2 [file mbio.03470-20-sf002.pdf]

**A**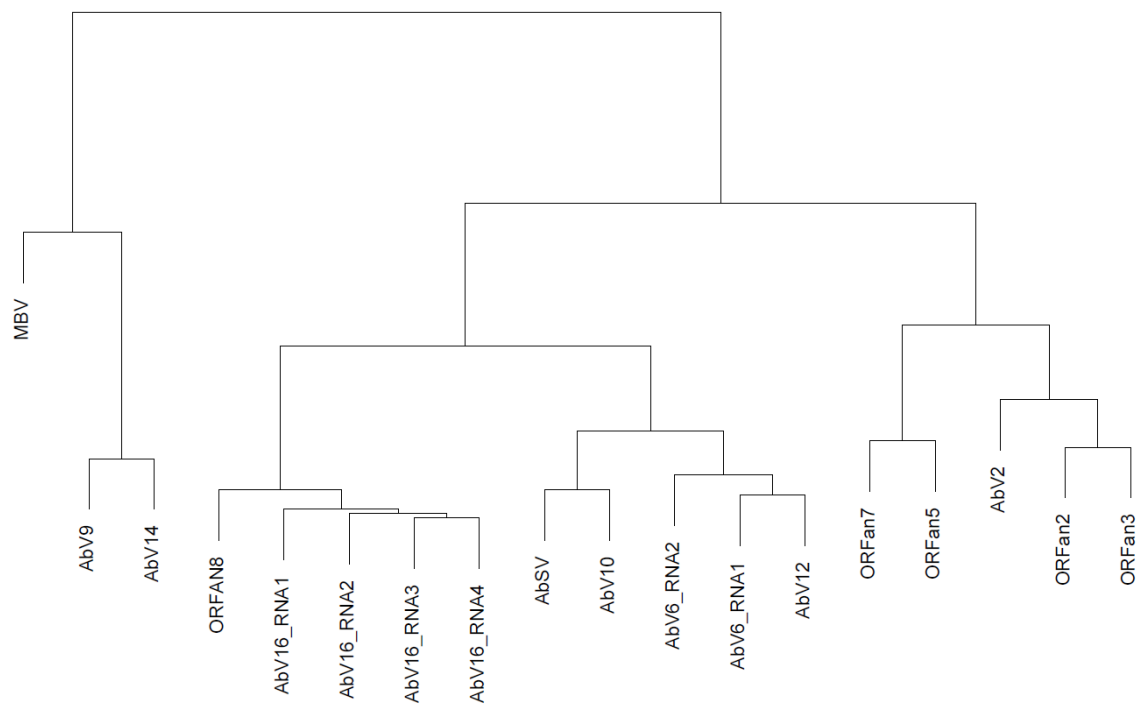**B**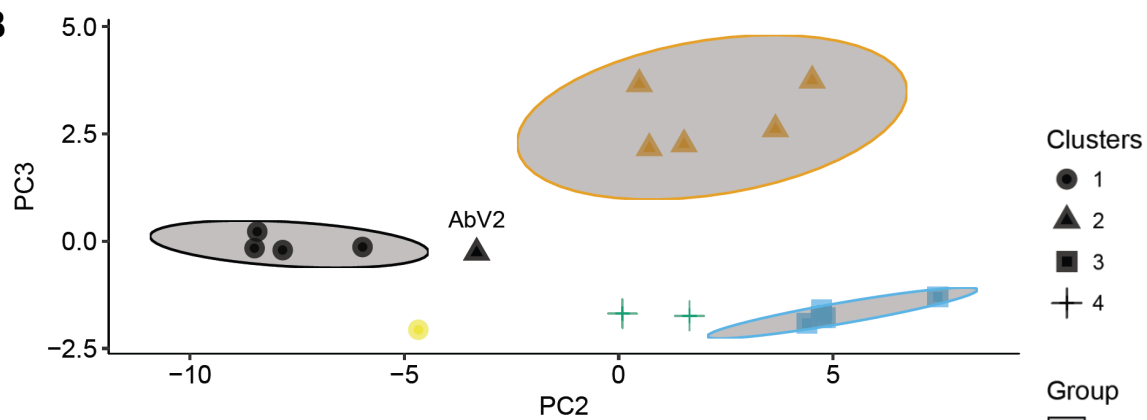**C**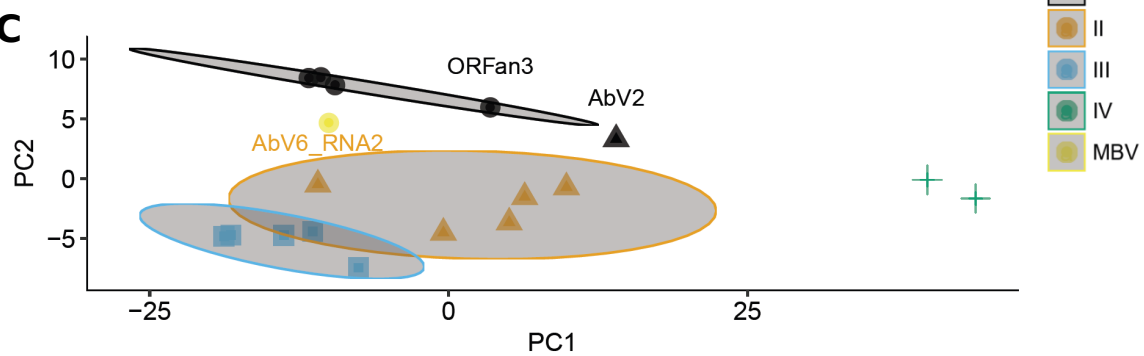

Supplement: FIG S3 [file mbio.03470-20-sf003.pdf]

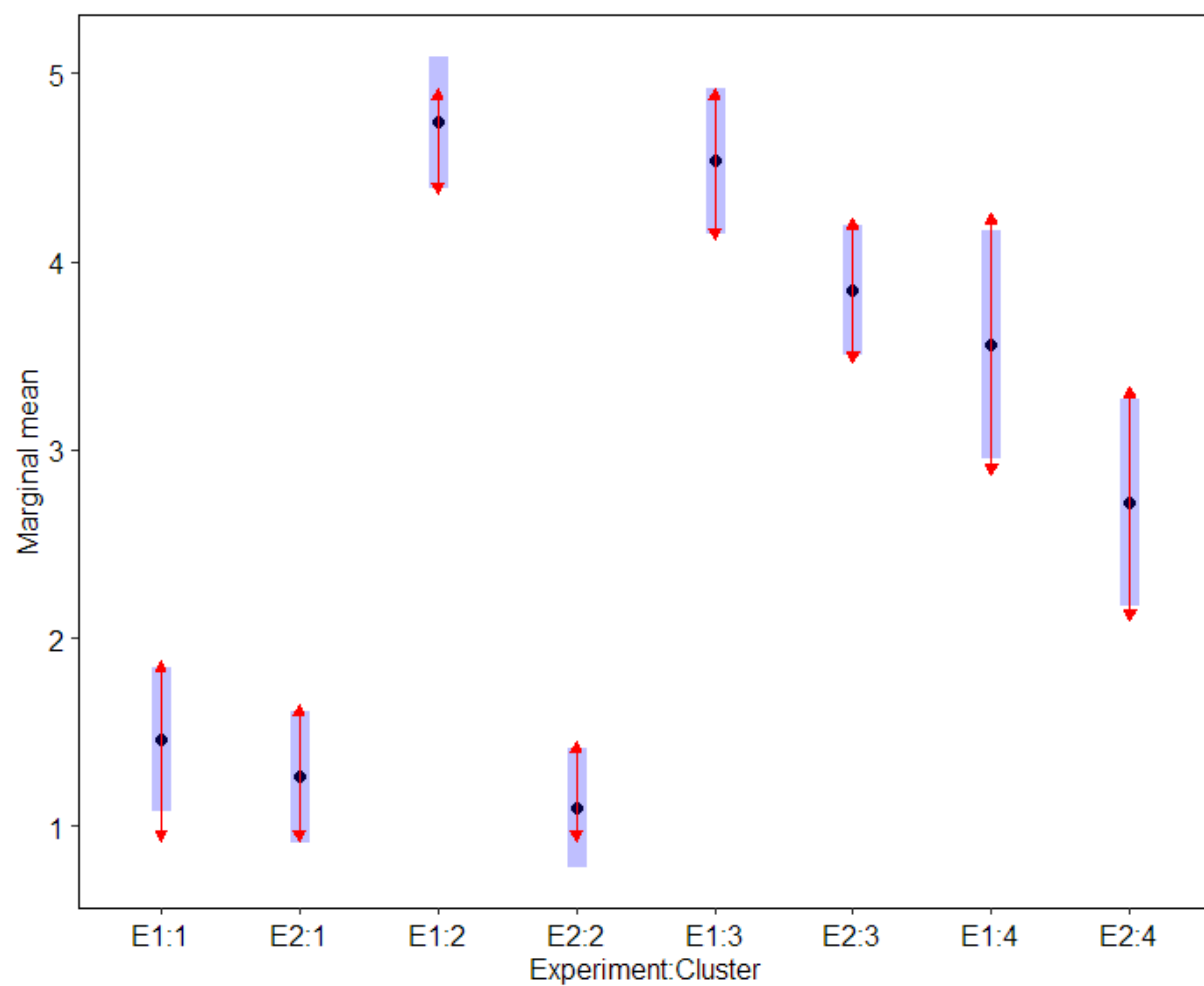

Supplement: FIG S4 [file mbio.03470-20-sf004.pdf]

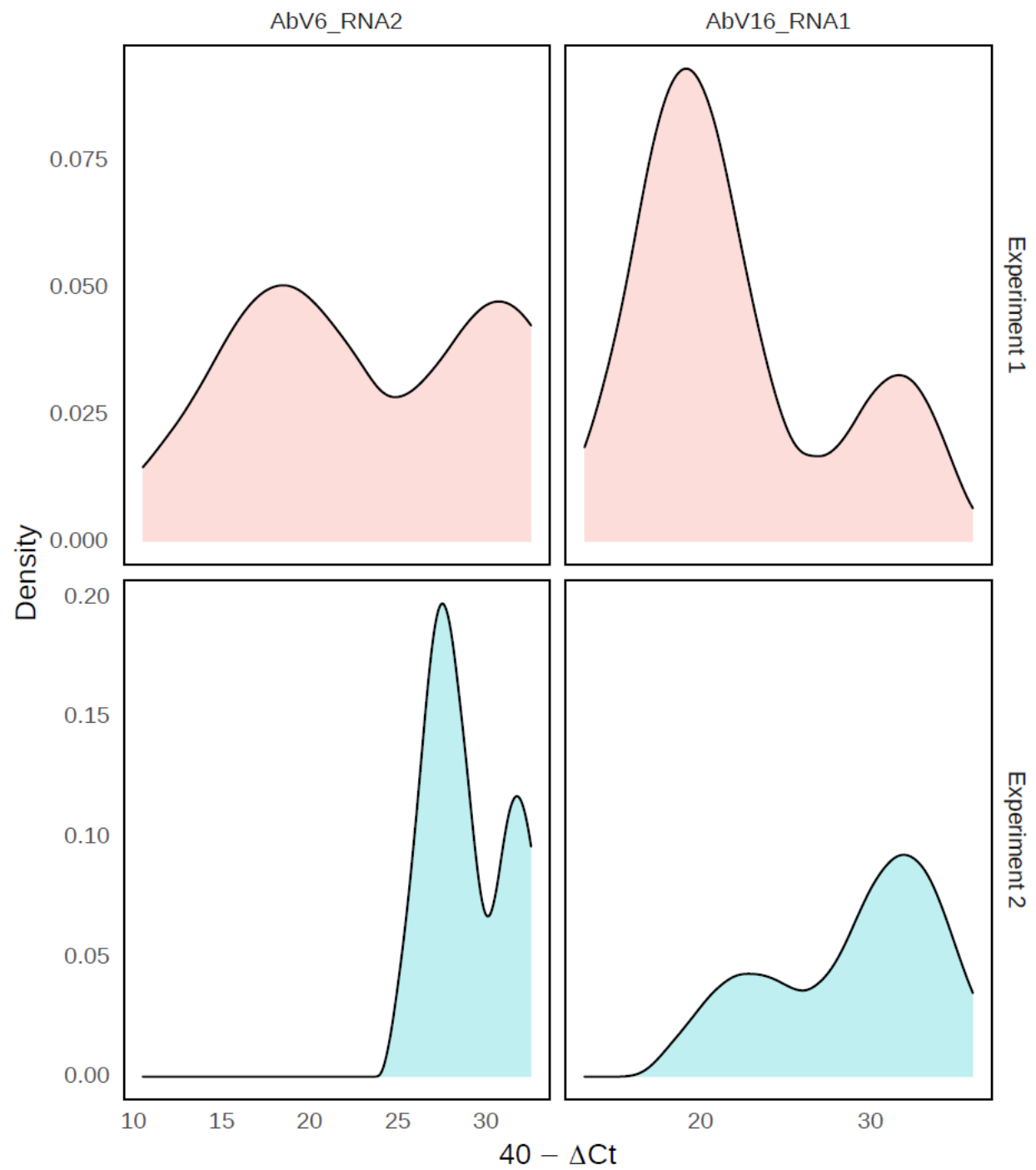

Supplement: FIG S5 [file mbio.03470-20-sf005.pdf]

Experiment 1

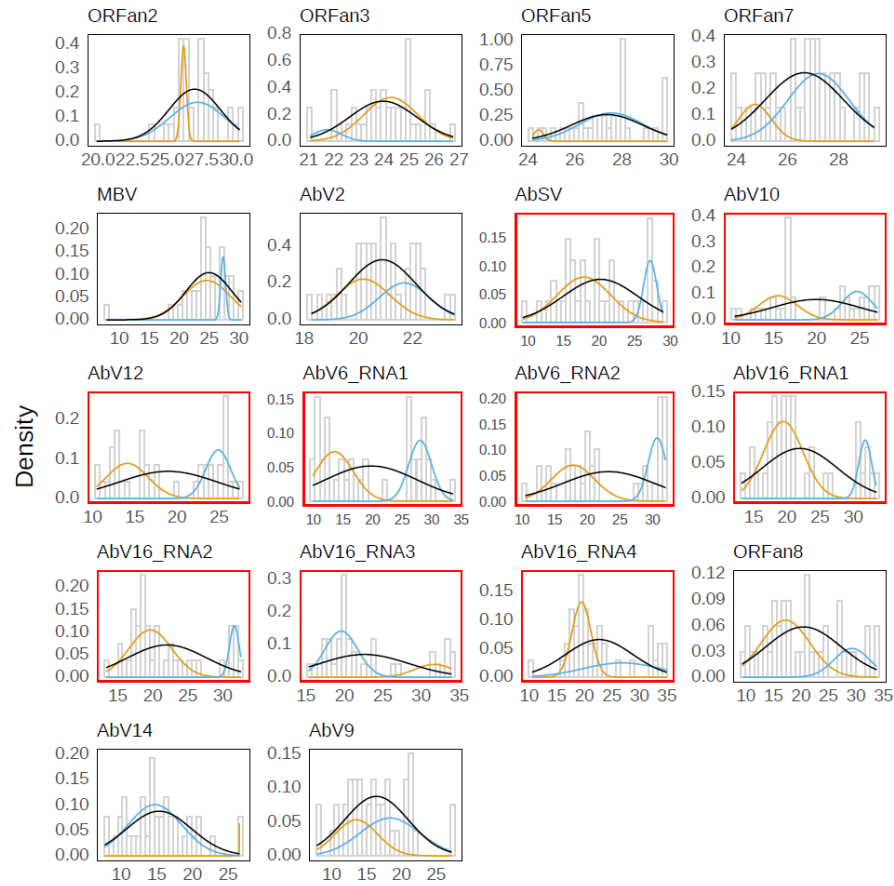

Experiment 2

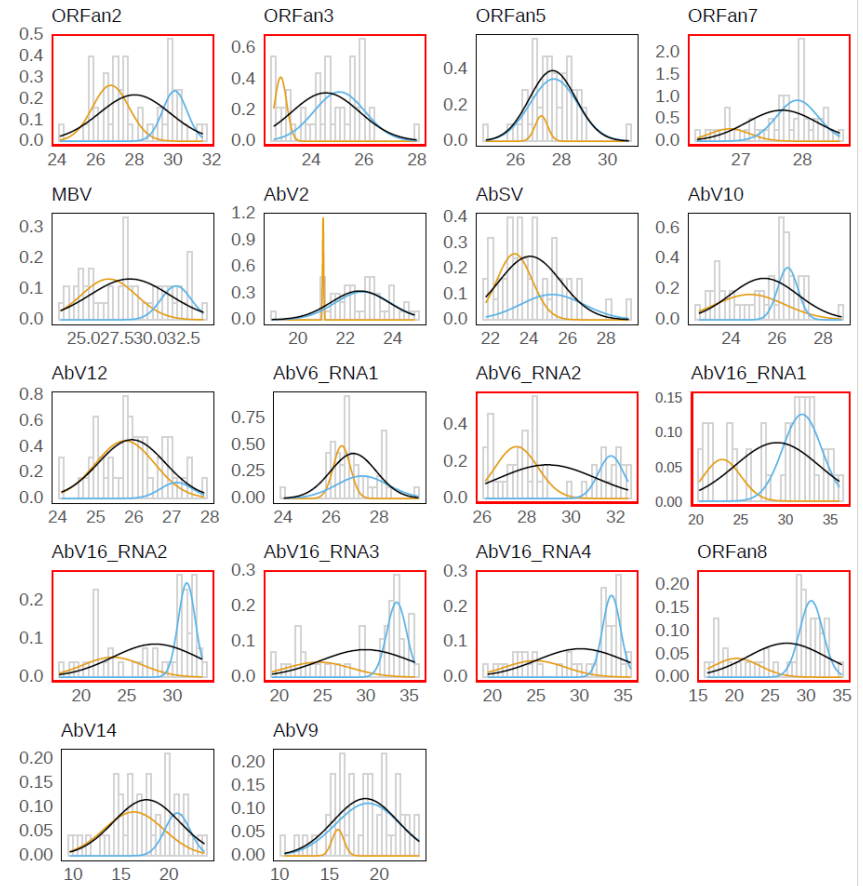

40 - ΔCt

Components — comp.1 — comp.2 — norm

Supplement: FIG S6 [file mbio.03470-20-sf006.pdf]
