# Supplementary material for: Viral Interactions and Pathogenesis during Multiple Viral Infections in Agaricus bisporus
Source: mBio. 2021 Feb 9;12(1):e03470-20. doi: 10.1128/mBio.03470-20 (PMC8545118; doi:10.1128/mBio.03470-20)
Supplement: TABLE S1 [file mbio.03470-20-st001.docx]

| Virus/ORFan name |  | Primer | Primer sequence | Product (bp) |
| --- | --- | --- | --- | --- |
| Agaricus bisporus virus 2 | Helicase | C1_A1_R | CTTTGACGCAGCCACAACTA | 126 |
|  |  | C1_A2_F | GCGTGACTCTGGCTAACCTC |  |
| Agaricus bisporus virus 11 |  | C10_A3_R | TCTCGATGGGCCTCAAAACC | 96 |
|  |  | C10_A4_F | TGCTGGCGACTTTTCTCAGT |  |
| Mushroom bacilliform virus |  | C11_A3_R | CCTGCTTTGTTGCTGCGATT | 138 |
|  |  | C11_A4_F | TCAGAGGCGTTGGAACCTTC |  |
| Agaricus bisporus virus 6 | RNA 2 | C12_q_1_F | CCGTACTCATCACGTGCAGT | 85 |
|  |  | C12_q_1_R | GCCTTCGAGATACAGCCTCC |  |
| Agaricus bisporus virus 9 | N-term | C13_A1_R | ACTGGTCTACGGCATCCTCT | 148 |
|  |  | C13_A2_F | AAGGATGGTGAAGCCCATGG |  |
| Agaricus bisporus virus 9 | RdRp | C14_A2_R | GATCGCCGGAGAACTGCATA | 123 |
|  |  | C14_A3_F | TCGACGCATGGCTTATGGAA |  |
| Agaricus bisporus virus 13 |  | C15_A3_R | AGAAGAAGGTGGAGTTGGCG | 114 |
|  |  | C15_A4_F | GCTGCCCGAATTGATGAACC |  |
| ORFan 2 |  | C17_A2_R | AGAAGTCGTGCAAGTGGGAG | 137 |
|  |  | C17_A3_F | CGAACGACGGGAACAACCTA |  |
| Agaricus bisporus virus 9 | Helicase | C18_A1_R | TCAGCCCTCTCCACGACATA | 89 |
|  |  | C18_A2_F | GAGGGTGTTGCTGCAGGTAT |  |
| ORFan 7 |  | C19_A1_R | ATTTCCCAGCATCCGTCAGG | 89 |
|  |  | C19_A2_F | CTACGGTCCGTCGAAAGGAG |  |
| Agaricus bisporus virus 6 | RNA 1 | C2_q_1_F | TGGGATGGCAGACTGATGTG | 125 |
|  |  | C2_q_1_R | CGGATCGGCCCTAGACAAAG |  |
| Agaricus bisporus virus 16 | RNA 2 | C20_q_1_F | CCGACAATGCGTGGTTGTTC | 149 |
|  |  | C20_q_1_R | GGGCCCTTCAAAGTCAGGTT |  |
| Agaricus bisporus virus 14 |  | C21_q_1_F | TTGCTCCCGATGAATCCTGG | 133 |
|  |  | C21_q_1_R | AAGTCAGTTCCGAGTCCACC |  |
| Agaricus bisporus virus 15 |  | C21a_q_1_F | CGTCTCCGAATGCAAGCATG | 144 |
|  |  | C21a_q_1_R | GATCGCGAACCCGTTCAATG |  |
| Agaricus bisporus virus 16 | RNA 1 | C22_q_1_F | ACATTATCGACGCCGCTCTT | 114 |
|  |  | C22_q_1_R | TTGCTTTCAGTCCGCAAAGC |  |
| Agaricus bisporus virus 2 | N-term | C23_A1_R | CTGCTTCCGTAAATGTCGCG | 98 |
|  |  | C23_A2_F | TTGGAGTCCCGTTGCGTTAA |  |
| ORFan 6 |  | C24_A2_F | ACCAGTGGTCTATCGAGGCT | 106 |
|  |  | C24_q_1_R | ACCTGAGAACCTTCCATGCG |  |
| ORFan 4 |  | C27_q_1_F | CGAAGAGGGGCATCCAAGTT | 114 |
|  |  | C27_q_1_R | TCACCAAGTACTTGTCCCGC |  |
| ORFan 3 |  | C28-C35_q_1_F | GAATGGGAGATTGCTTCGGC | 87 |
|  |  | C28-C35_q_1_R | ACTCCCTTCTCCTTCGTCGA |  |
| Agaricus bisporus virus 16 | RNA 3 | C29_q_1_F | TGCACGAAATCAGGAAGGCT | 146 |
|  |  | C29_q_1_R | ATAGGGCGGCATCTTAACGG |  |
| Agaricus bisporus virus 3 |  | C3_A2_R | CGCCTTACCACTGGAACATT | 117 |
|  |  | C3_A3_F | ATGCCTGAAAATCGTGATGG |  |
| Agaricus bisporus virus 16 | RNA 4 | C31_A1_F | GCTTCGGCCCACAAATCTCC | 150 |
|  |  | C31_q_A1_R | CCAGTCTTCCCCGATCAAGG |  |
| Agaricus bisporus virus 16 | RNA 5 | C33_A1-GOOD_F | GCTTCACGAACGCTGAAGTA | 146 |
|  |  | C33_q_A1_R | GCCGCTTACTGGACCACTTT |  |
| Agaricus bisporus virus 7 |  | C4_q_1_F | GTTCCACCAAACGCCACTTC | 123 |
|  |  | C4_q_1_R | AGCCCAGTGTAAATGAGGCC |  |
| Agaricus bisporus endornavirus 1 |  | C40_1_F | CTCACCGACCTTCGTTGACA | 93 |
|  |  | C40_q_1_R | TTTTCGTCAGCGCCTGTTTG |  |
| Agaricus bisporus mitovirus 1 |  | C41_A3_R | TGTTTTACTAGGAGACCAAGGCA | 135 |
|  |  | C41_A4_F | AGAGCTGGACTTAGATCCAGT |  |
| Agaricus bisporus spherical virus |  | C5_A6_R | CTGGTACCGGCCCATATGTC | 126 |
|  |  | C5_A7_F | AAGAGATGGCTATGGCGTGG |  |
| Agaricus bisporus virus 5 |  | C6_A1_R | ATCGTTTCACCTACGCCTCG | 107 |
|  |  | C6_A2_F | CCAAACTCTCGCCTACGTCA |  |
| Agaricus bisporus virus 10 |  | C7_A10_R | CACCAAGCCAAAATGTCGCA | 110 |
|  |  | C7_A11_F | CAAAGTTATGCGCGGCCTAC |  |
| Agaricus bisporus virus 8 |  | C8_A7_R | TAGGTACCCCAAGCCGATCA | 118 |
|  |  | C8_A8_F | CACCAACTTAGGCGTCGAGA |  |
| Agaricus bisporus virus 12 |  | C9_Con_4_R | GTGAAGTCCAGGAGGTTCGA | 107 |
|  |  | C9_Con_5_F | TTGTACATGCAGATGGTCGC |  |
