# Supplementary material for: Viral Interactions and Pathogenesis during Multiple Viral Infections in Agaricus bisporus
Source: mBio. 2021 Feb 9;12(1):e03470-20. doi: 10.1128/mBio.03470-20 (PMC8545118; doi:10.1128/mBio.03470-20)
Supplement: TABLE S2 [file mbio.03470-20-st002.docx]

|  | Experiment 1 | | Experiment 2 | |
| --- | --- | --- | --- | --- |
| RNA | ^†^Log lik | ^‡^*p* | ^†^Log lik | ^‡^*p* |
| ORFan2 | 2.35 | 0.883 | 17.34 | 0.014 |
| ORFan3 | 2.00 | 0.918 | 14.95 | 0.030 |
| ORFan5 | 1.69 | 0.933 | 4.17 | 0.664 |
| ORFan7 | 2.43 | 0.873 | 15.58 | 0.025 |
| MBV | 4.22 | 0.668 | 7.28 | 0.335 |
| AbV2 | 0.32 | 0.996 | 8.09 | 0.263 |
| AbSV | 15.26 | 0.034 | 6.46 | 0.410 |
| AbV10 | 21.46 | 0.003 | 10.45 | 0.133 |
| AbV12 | 31.68 | 0.000 | 0.57 | 0.991 |
| AbV6_RNA1 | 34.20 | 0.000 | 8.76 | 0.225 |
| AbV6_RNA2 | 35.03 | 0.000 | 36.49 | 0.000 |
| AbV16_RNA1 | 29.03 | 0.001 | 20.83 | 0.004 |
| AbV16_RNA2 | 31.31 | 0.001 | 43.30 | 0.000 |
| AbV16_RNA3 | 31.60 | 0.000 | 41.24 | 0.000 |
| AbV16_RNA4 | 21.24 | 0.005 | 45.20 | 0.000 |
| ORFan8 | 4.99 | 0.574 | 36.50 | 0.000 |
| AbV14 | 1.92 | 0.918 | 4.49 | 0.635 |
| AbV9 | 0.91 | 0.981 | 1.20 | 0.965 |

† Log likelihood ratio test statistic

‡ Probability calculated from 10 000 bootstrap comparisons of likelihood the viral abundances were drawn from multiple univariate distributions
