# Supplementary material for: Viral Interactions and Pathogenesis during Multiple Viral Infections in Agaricus bisporus
Source: mBio. 2021 Feb 9;12(1):e03470-20. doi: 10.1128/mBio.03470-20 (PMC8545118; doi:10.1128/mBio.03470-20)
Supplement: TABLE S3 [file mbio.03470-20-st003.docx]

|  | Experiment 1 | | Experiment 2 | |
| --- | --- | --- | --- | --- |
|  | r | *p* | r | *p* |
| Cluster 1 | -0.02 | 0.828 | 0.20 | 0.002 |
| Cluster 2 | 0.11 | 0.099 | -0.05 | 0.375 |
| Cluster 3 | 0.33 | <0.001 | 0.60 | <0.001 |
| Cluster 4 | 0.06 | 0.622 | 0.05 | 0.654 |
| ORFan2 | -0.15 | 0.365 | 0.04 | 0.783 |
| ORFan3 | -0.09 | 0.573 | 0.15 | 0.304 |
| ORFan5 | 0.02 | 0.884 | 0.02 | 0.913 |
| ORFan7 | 0.04 | 0.797 | -0.01 | 0.966 |
| MBV | 0.03 | 0.878 | 0.66 | <0.001 |
| AbV2 | 0.01 | 0.960 | -0.04 | 0.761 |
| AbSV | 0.17 | 0.294 | -0.15 | 0.294 |
| AbV10 | 0.17 | 0.306 | -0.01 | 0.931 |
| AbV12 | 0.18 | 0.272 | -0.05 | 0.736 |
| AbV6_RNA1 | 0.04 | 0.794 | -0.24 | 0.090 |
| AbV6_RNA2 | 0.10 | 0.558 | -0.08 | 0.564 |
| AbV16_RNA1 | 0.38 | 0.014 | 0.62 | <0.001 |
| AbV16_RNA2 | 0.35 | 0.028 | 0.61 | <0.001 |
| AbV16_RNA3 | 0.36 | 0.022 | 0.58 | <0.001 |
| AbV16_RNA4 | 0.36 | 0.023 | 0.58 | <0.001 |
| ORFan8 | 0.26 | 0.106 | 0.67 | <0.001 |
| AbV14 | 0.02 | 0.883 | 0.14 | 0.347 |
| AbV9 | 0.09 | 0.586 | -0.05 | 0.728 |
